# Outcome of children with panarteritis nodosa: a series of 30 cases

**DOI:** 10.1186/1546-0096-9-S1-P97

**Published:** 2011-09-14

**Authors:** E Merlin, R Mouy, P Quartier

**Affiliations:** 1 CHU Clermont-Ferrand, INSERM CIC 501, 63003 Clermont-Ferrand, France; 2 Hopital Necker, AP-HP, Paris, France

## Background

Childhood panarteris nodosa (PAN) is a rare vasculitis. Cutaneous and systemic forms are distinguished based on systemic involvement.

## Patients and methods

We recorded the data of children who were diagnosed as PAN in Necker Hospital between 1986 and 2006. Patients were classified as systemic PAN if at least one of the following organs was involved: central or peripheral nervous system, testis, kidney, lungs, heart or gastro-intestinal tract. Data are shown as median and range.

## Results

30 patients (20 girls, 10 boys) aged 7.2 years (2-14) were included. 29 had fever and all 30 had marked asthenia. The organs involved at diagnosis were: skin (24/30), muscle (12/30), nervous system (peripheral 4/30, lymphocytic meningitis 2/30), abdominal pain (6/30), kidney (2/30). Eleven children had an initial systemic presentation. All children had marked inflammatory syndrome. Sixteen had evidence of necrotizing panarteritis on histology, 14 had atypical features without necrosis. Angiography was performed in 2 patients and was normal. First-line therapy consisted in NSAID alone in 8 patients, IgIV in 4, steroids in 18. Overall, 6 patients did never need steroids, 8 patients needed cyclophosphamide. After 70 months of follow-up (7-178), 29 patients were alive, 24 disease-free (16 treatment-free), 5 with active disease, 1 patient died from sepsis. Patients with cutaneous form without histological necrosis had a better outcome. Four patients with initial cutaneous presentation subsequently developed systemic manifestations.

## Conclusion

The prognosis of pediatric PAN may be linked to both clinical and histological features.

